# TMEM176B Promotes EMT via FGFR/JNK Signalling in Development and Tumourigenesis of Lung Adenocarcinoma

**DOI:** 10.3390/cancers16132447

**Published:** 2024-07-03

**Authors:** Ping-Hui Sun, Siyu Xia, Runzhu Yuan, Bin Zhang, Guangsuo Wang

**Affiliations:** 1 Department of Thoracic Surgery, The Second Clinical Medical College of Jinan University, Shenzhen People’s Hospital, Shenzhen 518000, China; sunp2@cardiff.ac.uk (P.-H.S.); zhangbin@smail.nju.edu.cn (B.Z.); 2 Integrated Chinese and Western Medicine Postdoctoral Research Station, Jinan University, Guangzhou 510632, China; 3 Division of Cancer and Genetics, School of Medicine, Cardiff University, Cardiff CF14 4XN, UK; 4 Department of Reproductive Medicine, Dongguan Maternal and Child Health Care Hospital, Dongguan 523000, China; syxbestmayer@whu.edu.cn; 5 School of Medicine, The First Affiliated Hospital of Southern University of Science and Technology, Shenzhen People’s Hospital, Shenzhen 518000, China; 12331414@mail.sustech.edu.cn

**Keywords:** lung cancer, TMEM176B, EMT, cellular microenvironment, single-cell sequence, proteomics

## Abstract

**Simple Summary:**

TMEM176B has been reported to play a significant role in the regulation of dendritic cell maturation and other immune functions. Additionally, TMEM176B has been implicated in the development of various cancers, including breast cancer and colorectal cancer, where its expression levels correlate with disease progression and patient outcomes. This study aims to investigate the role of TMEM176B in the development of lung adenocarcinoma (LUAD). Our research indicates that the overexpression of TMEM176B is associated with a poor prognosis in LUAD patients, signifying more aggressive disease and reduced survival rates. Moreover, we have found that TMEM176B overexpression enhances the epithelial-mesenchymal transition (EMT) via the FGFR/JNK signalling pathway. This pathway is integral to cell growth, differentiation, and survival and its dysregulation can contribute to tumour progression and metastasis. These findings suggest that TMEM176B could serve as a potential therapeutic target for LUAD treatment, offering new avenues for developing targeted therapies.

**Abstract:**

Lung cancer, the leading cause of cancer-related incidence and mortality worldwide, is characterised by high invasiveness and poor prognosis. Novel therapeutic targets are required, especially for patients with inoperable metastatic disease requiring systemic therapies to improve patients’ welfare. Recently, studies indicated that TMEM176B is a positive regulator in breast and gastric cancers, and it could be a potential target for treatment. In this study, we used single-cell sequencing, proteomics, Co-IP, and in vivo and in vitro experimental models to investigate the role of TMEM176B in lung adenocarcinoma development. Our study indicated that TMEM176B expression was enhanced in lung adenocarcinoma tissues, and it was associated with shorter overall survival (OS). TMEM176B promoted cellular functions, including cell proliferation, invasion, migration and adhesion in vitro and tumour growth in vivo. Moreover, the tube formation ability of endothelial cells was enhanced by treating with the tumour cell-conditioned medium. We have also demonstrated that TMEM176B regulated EMT via the FGFR1/JNK/Vimentin/Snail signalling cascade. Overall, our study suggests TMEM176B could be a potential therapeutic target in lung adenocarcinoma.

## 1. Background

Lung cancer has consistently held the top position globally in terms of both incidence and mortality rates among all types of cancers. It is a malignant tumour with high invasiveness and poor prognosis, and the metastasis of cancer constitutes a pivotal stage in the progression of the disease. Epithelial mesenchymal transition (EMT) represents the critical stage in cancer metastasis. Tumour cells typically originate from epithelial cells that adhere tightly to each other. However, mesenchymal cells diminish epithelial cell characteristics, enhancing cell migration and invasion capabilities and facilitating the tumour’s movement to alternative locations. EMT enhances the plasticity of tumour cells, allowing primary tumour cells to infiltrate neighbouring tissues and undergo distant metastasis. EMT is not only vital in tumour progression but also holds significance in embryonic development. For example, neural crest cells undergo EMT to differentiate into various tissues and cells. The principal mechanisms revolve around the TGF-β, Wnt and FGF signalling pathways to regulate its downstream factors, including E-cadherin, Vimentin and MMPs, and transcription factors such as Twist, Snail and Slug [1,2].

Tumour cells predominantly regulate EMT through the TGF-β signalling pathway, with additional involvement from the PI3K, MAPK, Hedgehog and Wnt signalling pathways [3]. A recent study has shown that the knockout of Snail or Twist expression inhibits EMT in pancreatic cancer, resulting in the failure of cancer metastasis. Another study indicated that the knockout of Zeb1 expression results in the inhibition of EMT [4,5]. The TGF-β signalling pathway regulates EMT through the following two methods: (1) by modulating EMT-markers (EMT-Ms), via increasing Vimentin and decreasing E-cadherin expression, or (2) directly influencing EMT-transcription factors (EMT-TFs), such as Snail and Zeb2 [6,7]. Upon activation of the TGF-β signalling pathway, the transcription of downstream genes is regulated through histone modification. H2A.Z is the most crucial regulatory factor among EMT-Ms and reducing or inhibiting H2A.Z expression induces TGF-β-regulated EMT. The inhibition of epithelial gene expression was linked to the decrease in H2A.Z at the transcription start site (TSS) −1 nucleosome position, whereas the induction of mesenchymal gene expression relied on the elimination of H2A.Z at the TSS +2 nucleosome position [8]. TGF-β induces the expression of the Snail, and it requires the regulation of the Snail gene transcription in the TSS region via Smad2/Samd3 and CBP. The regulation of another EMT-TF, Zeb2, involves the participation of EZH2. This is achieved by decreasing the expression of EZH2 and H3K27me3 in the promoter region of Zeb2 to increase its expression [9,10].

Transmembrane protein 176B (TMEM176B) is a transmembrane protein, also known as LR8 or MS4B2 (membrane-spanning 4-domains subfamily B member 2). It was initially discovered in 1999, showing elevated expression in pulmonary fibroblasts and associated with lung fibrosis [11]. *Tmem176b* expression was associated with immunological tolerance [12]. Other studies have highlighted its role in the regulation of innate immunity, particularly in dendritic cell (DC) maturation and antigen presentation [13]. Studies indicate that TMEM176B is highly expressed in immature DCs compared to mature DCs; TMEM176B also regulates the acidification of endophagosomes via sodium channels [14,15]. Furthermore, TMEM176B knockout or the pharmacologic inhibition of TMEM176B demonstrates that it suppresses tumour growth through CD8+ T cells by triggering the activation of inflammasomes [14,16]. TMEM176B also plays an important role in the prognosis of colorectal cancer by enhancing the activation of the NLRP3 inflammasome [17]. Recent research suggests a rise in TMEM176B expression within breast cancer, notably in triple-negative breast cancer. Diminishing TMEM176B expression has been found to impede cell proliferation and migration in MDA-MB-231 cells, further affirming that decreased TMEM176B expression suppresses tumour growth in vivo. Treating MDA-MB-231 cells with TMEM176B antibodies also reduces cell proliferation, suggesting the potential of this gene as a target for breast cancer therapy [18]. 

Thus far, the abnormal expression of TMEM176B has been noted in gastric, prostate and breast cancer [18,19,20]. Nonetheless, its involvement in other cancer types remains predominantly unexplored. The current investigation seeks to elucidate the function of this molecule and its associated molecular mechanisms in lung adenocarcinoma utilising single-cell RNA sequencing, proteomics, as well as in vitro and in vivo experimental models.

## 2. Methods

### 2.1. Cell Lines and Cell Culture

The human lung adenocarcinoma cell lines, PC9 and A549, were sourced from the American Type Culture Collection (ATCC) in Gaithersburg, MD, USA. These cells were routinely cultured in the RPMI-1640 medium with 10% FBS and antibiotics at 37 °C with 5% CO_2_. HUVEC cells were procured from Fenghui Biotechnology (Changsha, China) and maintained in the human endothelial cell culture medium (Fenghui Biotechnology, Changsha, China) under similar conditions.

### 2.2. Human Lung Specimens

A lung adenocarcinoma tissue array was purchased from Outdo Biotech Co., Ltd. with ethical approval (HLugA180Su11, Shanghai, China). It included 90 paired tumours and adjacent normal tissues with data from routine follow-ups after surgery (Table S1).

### 2.3. Immunohistochemical Staining

Human lung adenocarcinoma tissue arrays and mouse CDX tumour tissues were paraffin-embedded and cut at a thickness of 3–4 μm using a microtome (Leica, Wetzlar, Germany). The sections were deparaffinised using xylene rehydrated with ethanol. The slides were incubated in 0.3% Triton until permeabilisation for 10 min, in a peroxidase suppressor (Thermo Scientific, Waltham, MA, USA) for 10 min, in a Citrate Buffer (pH 6.0) (Thermo Scientific, Waltham, MA, USA) for 45 min and then in a QuickBlock blocking buffer (Beyotime, Shanghai, China) for 20 min before being probed for primary antibodies (Table S2) for 1 h. After three washes, slides were treated with an HRP-conjugated secondary antibody, followed by exposure to the DAB substrate kit (Thermo Scientific, Waltham, MA, USA) for 5 min. A 1 min counterstain with Mayer’s haematoxylin ensued, followed by dehydration in ethanol and clearing in xylene before mounting.

### 2.4. Reverse Transcription-PCR

Total RNA was extracted from cultured cells using Trizol (Invitrogen, Waltham, MA, USA), followed by reverse transcription and PCR with PrimeScript™ RT Master Mix (Takara Bio USA Inc., San Jose, CA, USA). The TMEM176B primers (forward: 5′-ATGACGCAAAACACGGTGATT-3′, reverse: 5′-GCAGTTGTGTCAAAGCTGACT-3′) and GAPDH primers (5′-CTGGGCTACACTGAGCACC-3′ and 5′-AAGTGGTCGTTGAGGGCAATG-3′) were used under the conditions. The PCR products were separated using 1.5% agarose gels and photographed following staining with the SYBR Safe DNA nucleic acid gel stain (Invitrogen, Waltham, MA, USA).

### 2.5. Real-Time Quantitative PCR

The TMEM176B transcript levels in the cell lines were measured using real-time quantitative PCR (qPCR) with primer sequences identical to those used in reverse-transcription PCR. This was conducted with a StepOnePlus Real-Time PCR System (Applied Biosystems, Waltham, MA, USA), which is capable of simultaneously detecting 96 reactions. Transcript levels were determined relative to an internal standard and amplified concurrently with the samples.

### 2.6. Construction of Lentiviral Vectors and the Establishment of Corresponding Stable Transfectants

Lentiviral vectors were purchased from Hanbio Biotechnology (Shanghai, China), and the TMEM176B full sequence was cloned into a pHBLV-U6-MCS-CMV-ZsGreen-PGK-PURO vector. Cells were cultured with lentiviral vectors and 2 μg/mL of polybrene for 24 h and then replaced with a flash RPMI1640 medium. The verified overexpression transgenes and empty plasmids were transfected into PC9 (PC9^TMEM176Boe^ and PC9^ctrl^) and A549 (A549^TMEMB176Boe^ and A549^ctrl^) cells. Following a selection period using 2 μg/mL of puromycin (Gibco, Waltham, MA, USA), the confirmed transfectants were cultured in an RPMI-1640 medium supplemented with 0.2 μg/mL puromycin. 

### 2.7. Cell Function Assays

Cell proliferation, cell-matrix adhesion, cell invasion and cell migration assays were described in a previous report [21]; absorbance was measured using a spectrophotometer (SuPerMax 3100, Flash, Shanghai, China).

### 2.8. Immunoprecipitation (IP) and Western Bolt Analysis

In total, 1 × 10^6^ cells were cultured in a 25 cm^2^ flask overnight, and proteins were then extracted and quantified using a BCA protein assay kit (Thermo Scientific, Waltham, MA, USA). The protein samples were then incubated with a TMEM176B antibody (orb631961, Biorbyt, Cambridge, UK) at 4 °C for 1 h, followed by the addition of conjugated A/G protein magnetic beads (88802, Thermo Scientific, Waltham, MA, USA) and was further incubated for an hour. After two washes using a lysis buffer, the samples were boiled with a 2× sample buffer (S3401-10VL, Sigma-Aldrich Inc., Burlington, MA, USA). 

Proteins were separated by SDS-PAGE and transferred onto PVDF membranes (1214429, GVS North America, Sanford, ME, USA). They were then probed with primary and secondary antibodies (see Table S2), and protein bands were visualised using the chemiluminescence detection kit (Thermo Scientific, Waltham, MA, USA) and photographed using ImageQuant LAS 500 (GE HealthCare, Chicago, IL, USA).

### 2.9. In Vivo CDX Model

BALB/c nude mice were obtained from The GemPharmatech Co., Ltd. (Nanjing, China). Female mice aged 4 weeks, utilised in these investigations, were housed in microisolator cages within an SPF environment at the animal facility. Animal experiments were conducted in adherence to laboratory guidelines for animal care, and the protocols received approval from the IACUC of Shenzhen People’s Hospital. Tumour cells (1 × 10^6^ cells per mouse) were implanted into the subcutaneous flank tissue of BALB/c nude mice, and tumour size was measured using callipers twice a week. Upon reaching a diameter of 1.0–1.5 cm, mice were euthanised, and tumours were harvested for further experiments. Tumour volume = length × width^2^ × 0.5. 

### 2.10. Tube Formation Assay

Cells (40,000 per well) were placed in a 96-well plate pre-coated with Matrigel (500 µg per well). After 4 h of incubation, the cells were fixed with paraformaldehyde and captured under a microscope. The overall tube perimeter was assessed using ImageJ 1.53t software.

### 2.11. Single-Cell Libraries Construction and Sequencing

In total, 1 × 10^5^ PC9^ctrl^/PC9^TMEM176Boe^ cells and 1 × 10^5^ HUVEC cells were co-cultured in an ultra-low attachment plate (3473, Corning, Corning, NY, USA) for 24 h collected for Drop-seq sequencing. In short, the individual cells suspended in solution were combined with beads to co-encapsulate at an occupancy level of 0.05. Following the collection of individual droplets, mRNA was reverse-transcribed and amplified into cDNA. Afterwards, a 3′ gene expression library was generated using a Singleron Matrix Single Cell Processing System. Subsequently, sequencing was conducted on a NovaSeq 6000 (Illumina, San Diego, CA, USA) machine by Singleron (Nanjing, China).

### 2.12. Processing of scRNA-Seq Data

The raw sequencing reads obtained from cells was aligned to the pre-mRNA reference (Ensemble, mm10) and enumerated using a cell ranger (version 6.1.1) with default settings. Seurat (version 3.2.2) objects were created for each sample from the single-cell expression matrix, filtering out genes with fewer than 300 counts or a mitochondrial ratio exceeding 20%. The removal of doublets was carried out using DoubletFinder (version 2.0.2) with default settings, followed by data normalisation, dimensionality reduction, and cell clustering using Seurat. Clusters lacking specific marker genes and showing a relatively low gene content were excluded. The count matrix of each sample underwent normalisation using the “SCTransform” function. Variable genes were chosen and projected into a low-dimensional subspace using canonical correlation analysis (CCA) to address batch effects among different samples. Features and anchors for subsequent integration were identified utilising the “FindIntegrationAnchors” and “IntegrateData” functions, ensuring that the calculation was grounded on all necessary Pearson residuals. Following data integration and scaling, principal component analysis (PCA) was conducted using the “RunPCA” function, and the dataset was clustered utilising the “FindNeighbors” and “FindClusters” functions. Dimensionality reduction was accomplished utilising the “RunUMAP” function, and cell types were allocated to each cluster based on the abundance of known marker genes.

### 2.13. Proteomics Research and Bioinformatics Analyses

Lung adenocarcinoma cells (*n* = 3 per group) were collected, and label-free quantitative proteomics technology established by Biotree Biomedical Technology Co., Ltd. (Shanghai, China) was employed.

Statistical analysis of the proteomic data was conducted, and the proteins showing differential abundance were subsequently refined based on criteria including unique peptides ≥ 1, foldchange ≥ 1.2 or ≤0.83 and *p* < 0.05. 

### 2.14. Gene Ontology (GO) Enrichment Analysis

We conducted GO enrichment analysis using clusterProfiler. Representative terms were chosen based on an adjusted *p* value cutoff of <0.05 and were visualised using the ggplot2 R package (version 3.3.2).

### 2.15. Cell–Cell Communication Inference

We used CellChat (version 1.1.3) to analyse the communication between cells through the networks of ligand–receptor interactions. This involved integrating a carefully curated database of ligand–receptor interactions with gene expression data, social network analysis, manifold learning and pattern recognition. We developed a model to estimate the probability of communication between different cell types and identified specific ligand–receptor pairs that represented this communication, using a significance cutoff of *p* value < 0.01.

### 2.16. Statistical Analysis

Statistical analysis was conducted using GraphPad Prism (Boston, MA, USA). One-way ANOVA or two-way ANOVA was employed for the analysis of all data, and survival was assessed using Kaplan–Meier survival analysis. A significance threshold of *p* < 0.05 was utilised for statistical significance determination.

## 3. Results

### 3.1. TMEM176B Expression Is Associated with Tumour Stages, Prognosis and Survival

**Figure 1:**
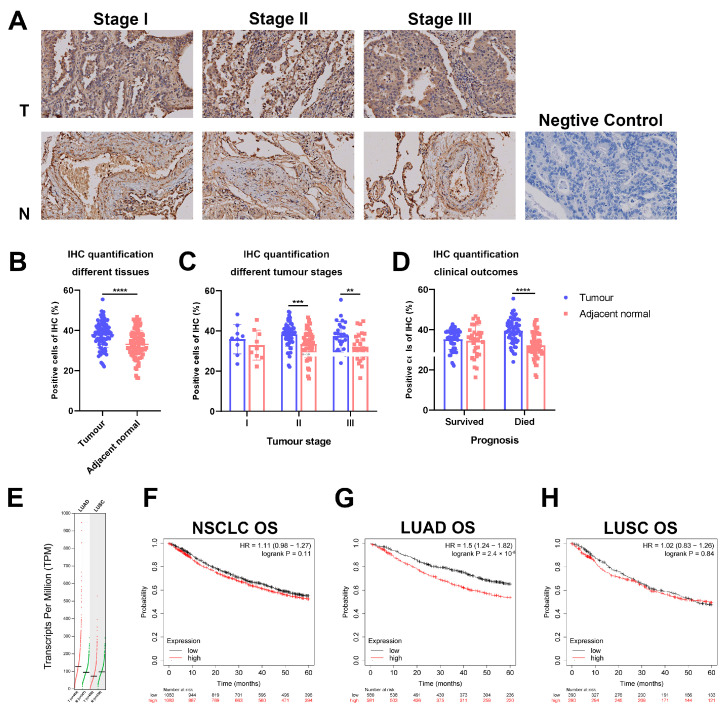
Expression of TMEM176B in lung cancer and clinical outcomes. (**A**) Tissue array IHC staining (400×, scale bar = 20 μm). The relative intensity of IHC staining was analysed using ImageJ software for comparisons with (**B**) tumour and adjacent normal tissues, (**C**) different tumour stages and (**D**) patients who survived or died from the disease. (**E**) TMEM176B expression in LUAD and LUSC (http://gepia2.cancer-pku.cn, accessed on 3 December 2023). Overall survival of TMEM176B in (**F**) NSCLC, (**G**) LUAD and (**H**) LUSC (https://kmplot.com/analysis/, accessed on 4 December 2023). **, *p* < 0.01, ***, *p* < 0.001 and ****, *p* < 0.0001.

The immunohistochemical analysis of tissue arrays demonstrated enhanced TMEM176B staining in tumour tissues as opposed to their corresponding adjacent normal tissues (Figure 1A). Further analysis of staining intensity affirmed elevated TMEM176B expression in lung adenocarcinoma (Figure 1B). These findings align with the results from the TCGA/GTEx database (Figure 1E), indicating heightened TMEM176B expression in lung adenocarcinoma (LUAD) and a slight decrease in expression in squamous cell carcinoma (LUSC). Furthermore, TMEM176B expression exhibited a significant increase in tumour stages II and III but not in tumour stage I (Figure 1C). This suggests that TMEM176B may play an important role in the development of lung adenocarcinoma. Additionally, we examined the associations of TMEM176B with prognosis and survival, revealing a significant enhancement in TMEM176B expression among patients who died from cancer (Figure 1D). The Kaplan–Meier survival curve was employed to assess the overall survival of patients. The TMEM176B expression level did not affect the survival of patients with NSCLC (Figure 1F) or squamous cell carcinoma (Figure 1H). Nevertheless, it was observed that in lung adenocarcinoma (Figure 1G), patients with elevated TMEM176B transcript levels experienced a significantly shorter overall survival (*p* = 2.4 × 10^−5^) compared to those with lower transcript levels.

### 3.2. Overexpression of TMEM176B in Lung Adenocarcinoma Cell Lines and Its Impact on In Vitro, In Vivo and Endothelial Cell Tube Formation

**Figure 2:**
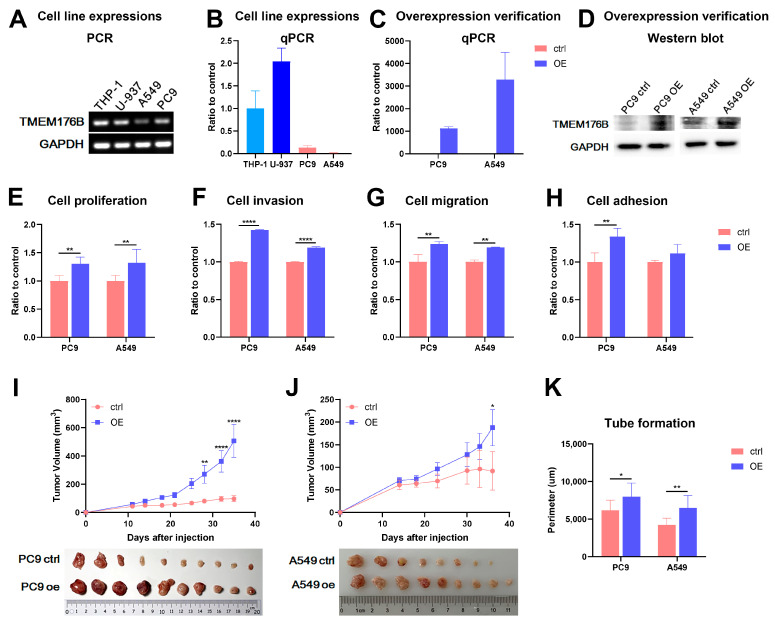
TMEM176B overexpression in in vitro and in vivo experiments. Examining TMEM176B expression in different cell lines using (**A**) PCR and (**B**) qPCR. Verification of TMEM176B overexpression using (**C**) qPCT and (**D**) Western blot. In vitro cell function assays for (**E**) cell proliferation, (**F**) cell invasion, (**G**) cell migration and (**H**) cell adhesion; overexpression of TMEM176B promoted all cell functions significantly. Establishing in vivo CDX mouse models (*n* = 10 per group) using (**I**) PC9 cells (control vs. overexpression: *n* = 10/10 vs. *n* = 10/10) and (**J**) A549 cells (control vs. overexpression: *n* = 8/10 vs. *n* = 9/10); overexpression of TMEM176B increased tumour growth in both cell lines significantly. (**K**) Tube formation assay of HUVEC cells was enhanced when the cells were treated with conditioned medium collected from PC9 or A549 cell lines. *, *p* < 0.05, **, *p* < 0.01 and ****, *p* < 0.0001.

The assessment of TMEM176B mRNA expression in cell lines was conducted through PCR (Figure 2A) and qPCR (Figure 2B). Notably, TMEM176B exhibited relatively lower expression in lung adenocarcinoma cell lines, specifically PC9 and A549, when compared with monocyte cell lines such as THP-1 and U-937. The overexpression of TMEM176B was achieved through a lentiviral vector carrying the full human TMEM176B sequence. This process was carried out using two lung adenocarcinoma cell lines, PC9 and A549, both of which displayed low TMEM176B expression levels (Figure 2A,B). The verification of TMEM176B overexpression in the transfectants was established through qPCR (Figure 2C) and Western blot analyses (Figure 2D). Elevated TMEM176B expression was evident in both PC9^TMEM176Boe^ and A549^TMEM176Boe^ cells, which were transfected with lentiviral vectors in comparison to their respective controls.

The impact of TMEM176B overexpression on various in vitro cell functions, including cell proliferation, invasion, migration and adhesion, was examined. The overexpression of TMEM176B in both PC9^TMEM176Boe^ and A549^TMEM176Boe^ cells significantly affected in vitro cell growth, with both cell lines exhibiting a promoted growth rate on day 3 compared to the empty plasmid control (Figure 2E). In invasion assays, both PC9^TMEM176Boe^ and A549^TMEM176Boe^ cells demonstrated an enhanced invasive capacity compared to the controls (Figure 2F). The overexpression of TMEM176B also exerted a notable influence on cell migration in both PC9^TMEM176Boe^ and A549^TMEM176Boe^ cells, as evidenced by a significantly increased motility compared to the empty vector control (Figure 2G). Finally, in PC9 cells, the overexpression of TMEM176B led to increased cell-matrix adhesion compared to PC9^ctrl^ (Figure 2H).

The overexpression of TMEM176B not only affected cellular functions but also had an impact on in vivo tumour growth in CDX models. The overexpression of TMEM176B significantly increased tumour growth in both PC9^TMEM176Boe^ (506.96 ± 115.15 mm^3^, *n* = 10/10 vs. 97.13 ± 21.78 mm^3^, *n* = 10/10; Figure 2I) and A549^TMEM176Boe^ (187.67 ± 39.91 mm^3^, *n* = 9/10 vs. 90.94 ± 41.99 mm^3^, *n* = 8/10; Figure 2J) cells compared to the empty vector controls. Additionally, it stimulated tube formation in HUVEC cells when treated with a conditioned medium from cells overexpressing TMEM176B (Figure 2K). The total tube perimeter was 7980.78 ± 1711.93 µm for PC9^TMEM176Boe^ cells compared to its control cells (6177.40 ± 1260.27 µm) and 6480.27 ± 1539.54 µm for A549^TMEM176Boe^ cells compared to its control cells (4229.19 ± 842.17 µm).

### 3.3. Unveiling the Cellular Landscape of Cancer Cell and Endothelial Cells Samples via Single-Cell Profiling

Single-cell RNA sequencing (scRNA-seq) was performed on a co-culture of PC9^TMEM176Boe^/PC9^ctrl^ and HUVEC samples. There were 6 samples in total, which included three samples co-cultured with PC9^TMEM176Boe^ (referred to as the “Case”) and three samples co-cultured with PC9^ctrl^ (referred to as the “Cont”).

**Figure 3:**
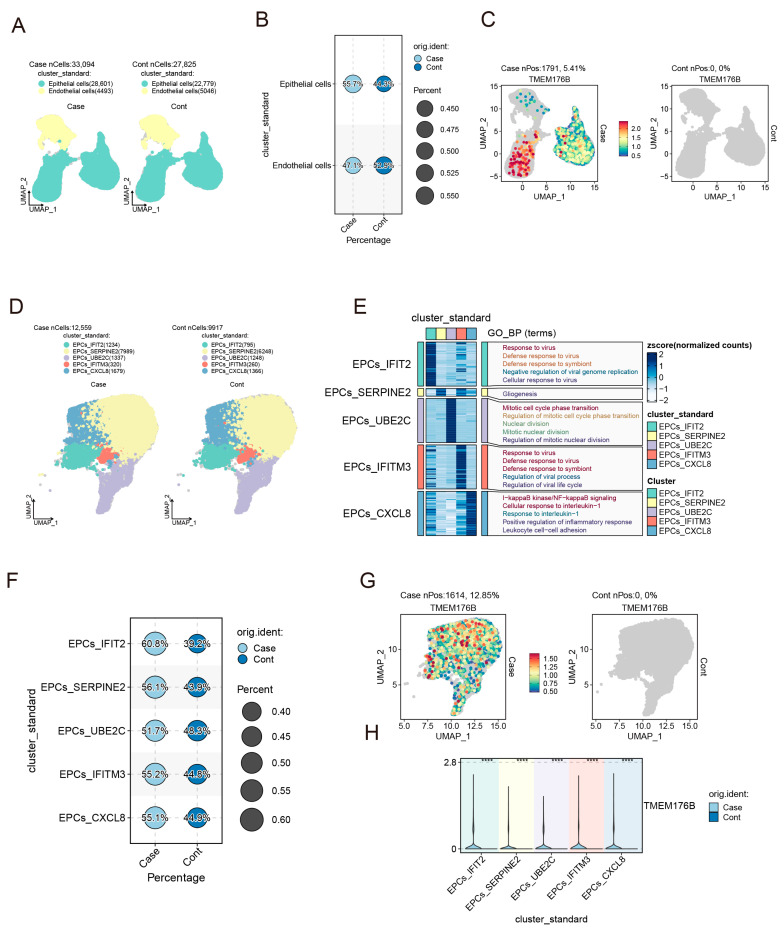
Cell population of co-cultured PC9 and HUVEC cells. (**A**) UMAP of total cells. (**B**) Percentage of cell compositions. (**C**) TMEM176B expression patterns in Case and Cont. (**D**) Subgroup of epithelial cells and their major GO term (**E**). (**F**) Composition percentage of each subgroup. (**G**) TMEM176B expression in the subgroup of epithelial cells. (**H**) The expression percentage of TMEM176B in the epithelial subgroup. ****, *p* < 0.0001.

After implementing rigorous cell filtration procedures, we successfully generated a comprehensive dataset consisting of 54,000 high-quality cellular transcriptomes derived from these tissues. To comprehensively visualise the global composition of cell types within cells, we employed the uniform manifold approximation and projection (UMAP) algorithm in our dataset. The results revealed the general component of cancer epithelial and endothelial cells in Figure 3A,B; it also indicated TMEM176B expression levels in different types of cells (Figure 3C). Moreover, it revealed the presence of five distinct cell types (Figure 3D) in epithelial cells and their representative GO term in each subgroup (Figure 3E,F). Interestingly, we observed significant alterations of TMEM176B in the number and distribution of cell types between the Case and Cont groups (Figure 3G) and shown graphically (Figure 3H).

### 3.4. Intercellular Crosstalk between TMEM176B Overexpression Cells and Endothelial Cells

**Figure 4:**
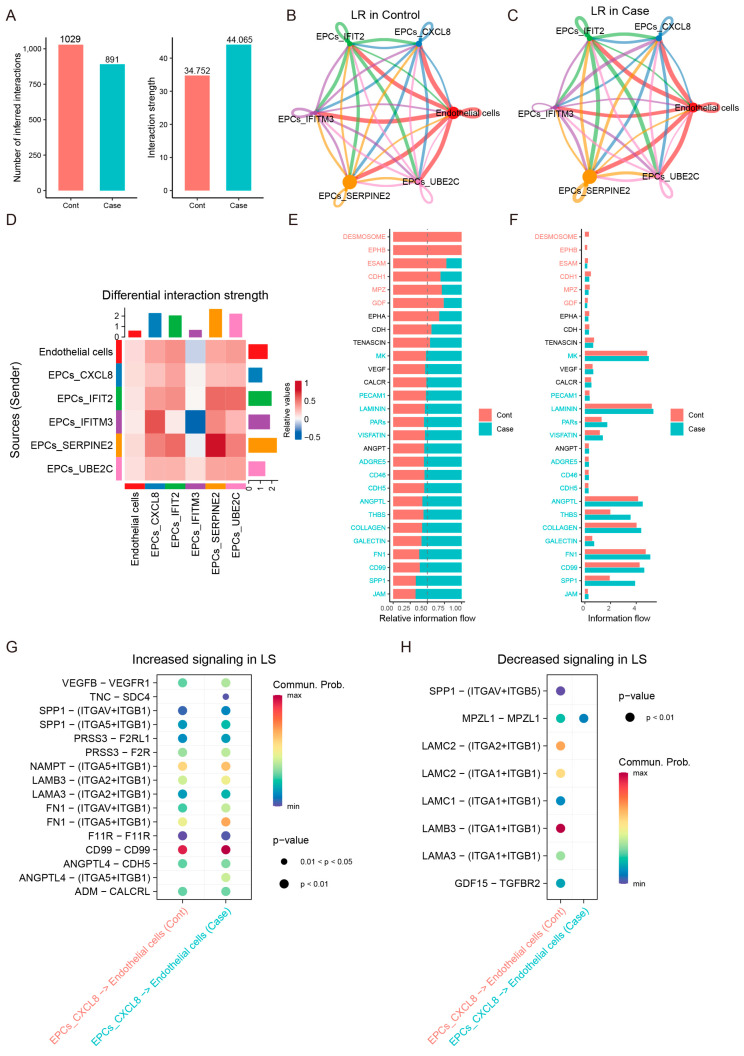
Ligand–receptor interactions between cancer cells and endothelial cells. (**A**) Overall interaction. Ligand–receptor interactions among endothelial cells and 5 subgroups of cancer cells in Cont (**B**) and Case (**C**). (**D**) The strength of interactions. (**E**) Alternative genes of interactions and their expression (**F**). Ligand–receptor pairs between the CXCL8 subgroup and endothelial cells were up-regulated (**H**) and down-regulated (**G**).

In order to elucidate the cellular crosstalk between cancer cells and endothelial cells, we performed cell–cell communication analysis using CellChat (Figure 4A). The results indicated that the most affected cellular ligand–receptor interactions occurred between cancer cells and endothelial cells, and TMEM176B overexpression enhanced the interaction strength (Figure 4B,C). These ligand–receptor interactions were primarily involved in JAM, SPP1, gelatin and ECM, including fibronectin and collagen (Figure 4E,F); these molecules play a crucial role in the regulation of EMT in cancers. We were particularly interested in the interactions between the CXCL8 subgroup of cancer cells and endothelial cells, where we demonstrated the further analysis of ligand–receptor interactions for these cells. It indicated that SPP1 and several ECMs, such as laminins and integrins, were up-regulated (Figure 4G) and down-regulated (Figure 4H) in these cells.

### 3.5. FGFR1 and Vimentin Interacted with TMEM176B

**Figure 5:**
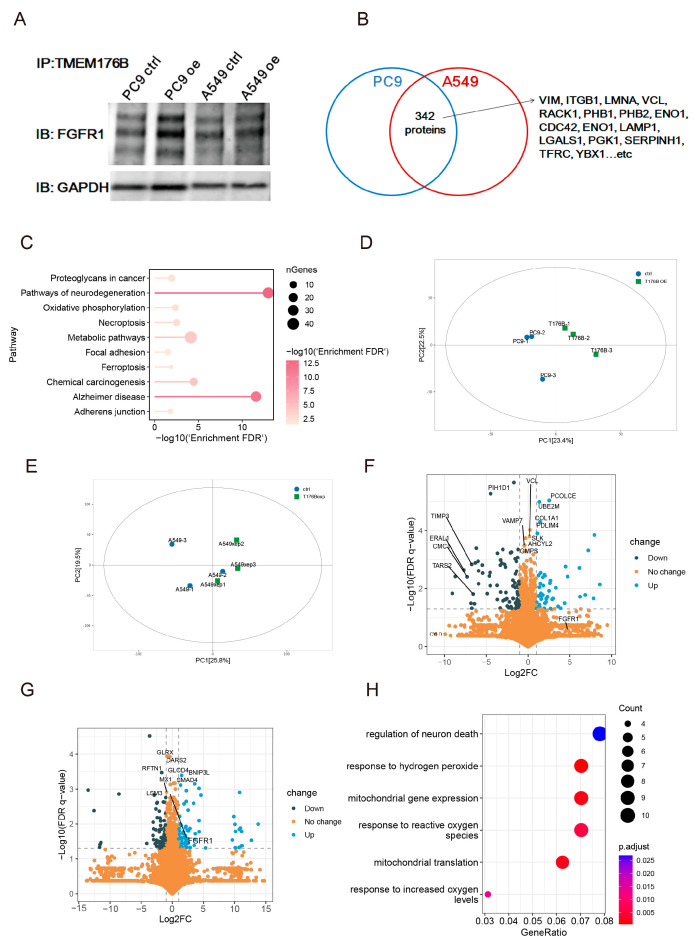
FGFR1 and Vimentin interacted with TMEM176B. (**A**) FGFR1 interacted with TMEM176B using Co-IP. (**B**) TMEM176B interacted with 342 proteins using mass spectrometry. (**C**) GO enrichment analysis of interacted proteins. Principal component analysis of proteomics in PC9 (**D**) and A549 (**E**) cells. Volcano plot analysis of proteomics in PC9 (**F**) and A549 (**G**) cells. (**H**) GO enrichment analysis for DEGs in PC9 cells.

To investigate the protein–protein interactions between TMEM176B and other proteins, immunoprecipitation (IP) was employed to identify potential candidates. Initially, Co-IP revealed the interaction between FGFR1 and TMEM176B (Figure 5A). Subsequently, proteins were purified from a PAGE gel using mass spectrometry, and we identified 342 proteins (Table S3) that interacted with TMEM176B, including Vimentin (Figure 5B,C). Proteomics analysis was conducted on PC9 and A549 cells (*n* = 3 for each group), and principal component analysis (PCA) results are presented in Figure 5D (PC9 cells) and Figure 5E (A549 cells). The findings indicate an enhanced FGFR1 level following TMEM176B overexpression, with a significant increase observed in A549^TMEM176Boe^ cells (Figure 5G). Moreover, in PC9^TMEM176Boe^ cells, the FGFR1 expression level was 20 times higher than that in PC9^ctrl^ cells, although statistical significance was not reached due to sample size limitations (Figure 5F). GO annotation enrichment analysis for DEGs in the PC9 cell is depicted in Figure 5H, while for the A549 cell, it did not reach a statistically significant level.

### 3.6. Involvement of FGFR/JNK/Vimentin Pathway in Regulation of Cell Functions by TMEM176B Overexpression

**Figure 6:**
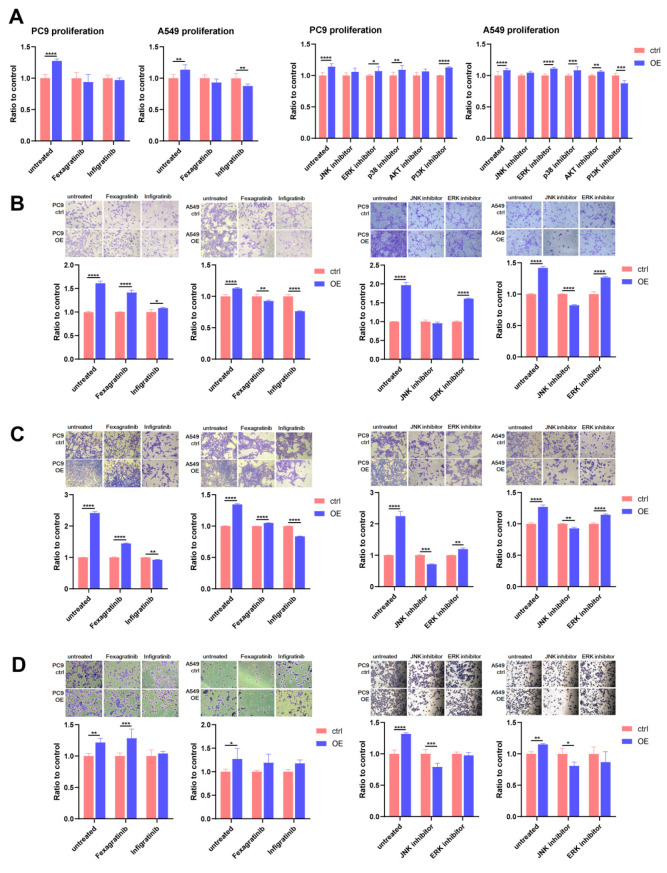
Overexpression of TMEM176B-enhanced cell functions in lung adenocarcinoma through FGFR and JNK. Cell function assays for PC9 and A549 treated with FGFR, JNK, ERK, p38, AKT or PI3K inhibitors; cell proliferation (**A**), cell invasion (**B**), cell migration (**C**) and cell adhesion (**D**). A decreasing trend was observed for all cell functions using FGFR inhibitors; there was also a significant reduction in cell functions when cells were treated with the JNK inhibitor. *, *p* < 0.05, **, *p* < 0.01, ***, *p* < 0.001 and ****, *p* < 0.0001.

To explore the signalling pathway in lung adenocarcinoma cells after TMEM176B overexpression, we chose candidates from our scRNA-seq and proteomics results and literature reviews. We introduced 5 nM of Fexagratinib (also known as AZD4547, MCE, USA) or Infigratinib (also known as BGJ-398, MCE, Monmouth Junction, NJ, USA) to treat PC9 and A549 cells, both of which are FGFR small inhibitors. As depicted in Figure 6, PC9^TMEM176Boe^ cells exhibited a significant reduction in cell proliferation (Figure 6A), invasion (Figure 6B), migration (Figure 6C) and adhesion (Figure 6D) when exposed to Infigratinib, whereas only a decrease in cell proliferation and migration was observed with Fexagratinib treatment. In the case of A549^TMEM176Boe^ cells, treatment with both inhibitors led to a pronounced decrease in all cell functions. The reductions in cell functions were more substantial with Infigratinib, and it even reversed the observed trend. 

Given the association between FGFR and Vimentin observed in previous experiments, we identified five potential candidates from the literature that could potentially participate in the regulation of this mechanism. We introduced 0.5 µM of JNK (SP600125, Selleck, Houston, TX, USA), ERK (FR180204, Selleck, USA), p38 (SB203580, Selleck, USA), AKT (GSK690693, Selleck, USA), or PI3K (PI-103, Selleck, USA) inhibitors to assess their impact on cell proliferation initially. The results revealed that the treatment of TMEM176B overexpression cells with the JNK inhibitor significantly attenuated the promotion of cell proliferation (Figure 6A). For other cell function assays, we employed JNK or ERK inhibitors to treat cells, and the findings suggested that JNK plays a pivotal role in regulating the EMT of TMEM176B overexpression cells. Specifically, the overexpression cells treated with the JNK inhibitor exhibited a reduction in enhancements of cell invasion (Figure 6B), migration (Figure 6C) and adhesion (Figure 6D) in both PC9 and A549 cells.

### 3.7. TMEM176B Regulates EMT via FGFR/JNK/Vimentin/Snail Signalling Cascade in Lung Adenocarcinoma

**Figure 7:**
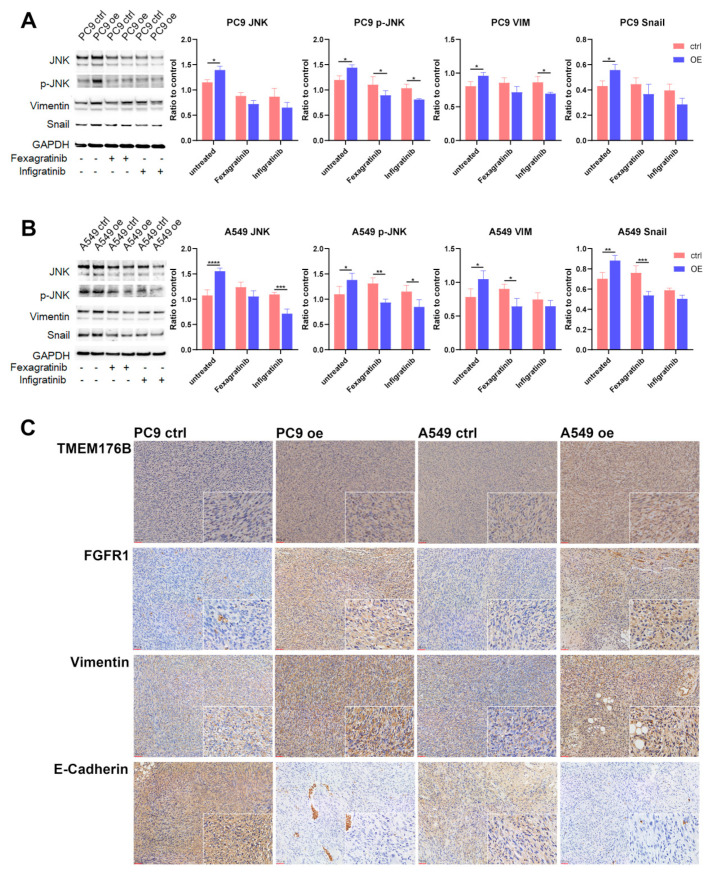
Overexpression of TMEM176B-enhanced EMT in lung adenocarcinoma via FGFR/JNK/VIM/Snail signalling cascade. Western blot and relative intensity of bands for PC9 (**A**) and A549 (**B**) cells. (**C**) IHC staining using mouse CDX tumour tissues (200× and 400×, scale bar = 50 μm). *, *p* < 0.05, **, *p* < 0.01, ***, *p* < 0.001 and ****, *p* < 0.0001.

To validate our hypothesis regarding the epithelial–mesenchymal transition (EMT) pathway, cells were treated with FGFR inhibitors, and the protein expression levels of JNK, p-JNK, ERK1/2, p-ERK1/2, Vimentin, Snail, Slug and the housekeeping protein GAPDH were examined in PC9 (Figure 7A) and A549 (Figure 7B) cells. The results indicate a significant increase in JNK and p-JNK protein levels, particularly in PC9 cells compared to A549 cells. Furthermore, the levels of JNK and p-JNK in TMEM176B overexpression cells were reduced to a comparable level when treated with FGFR inhibitors and downstream molecules, such as Vimentin and Snail, exhibited a similar trend with JNK, but there was no significance with ERK1/2, p-ERK1/2 and Slug (Figure S1). Furthermore, IHC staining results demonstrated that TMEM176B, FGFR1 and Vimentin were increased significantly in both overexpression PC9 and A549 cells and that E-cadherin was decreased in overexpression cells (Figure 7C). These findings suggest that TMEM176B is involved in enhancing EMT in lung adenocarcinoma through the FGFR/JNK/Vimentin/Snail axis.

## 4. Discussion

TMEM176A and TMEM176B are transmembrane proteins with involvement in the regulation of diverse cellular processes. Both TMEM176A and TMEM176B have been identified as negative regulators of dendritic cell (DC) maturation. Their expression is down-regulated subsequent to DC maturation, contributing to maintaining the immature state of the DCs [15,22]. Moreover, TMEM176A and TMEM176B have been demonstrated to have a role in the regulation of cancer pathology. The expression of these proteins is elevated in lymphoma, and notably, TMEM176A shows a significant increase in expression in lung cancer [23]. Both gene and protein expressions of TMEM176A are raised in glioblastoma, and it promotes the cell cycle via the ERK1/2 signalling pathway [24]. Nevertheless, TMEM176A has been identified as a tumour suppressor in pancreatic cancer, primarily regulated through the ERK signalling pathway [25]. Through literature reviews, it has been observed that TMEM176A assumes diverse roles across various types of cancers. It may function as either a positive or negative regulator, similar to the situation observed for TMEM176B in the context of cancers. Using transcriptome sequencing and conducting enrichment analysis demonstrated an association between TMEM176B and the differentiation of lung squamous cell carcinoma (GO:0030154) [26]. In gastric cancer patients, TMEM176B expression was observed to be elevated in tumour tissues compared to normal tissues and patients with a high TMEM176B expression exhibited significantly lower survival rates than those with a low expression [19]. Moreover, a recent study indicated an increase in TMEM176B expression in breast cancer and a reduction in TMEM176B expression suppressed tumour growth in vitro and in vivo [18]. In contrast, TMEM176B is a negative regulator in prostate cancer where the overexpression of TMEM176B reduces LNCap cell proliferation, invasion and migration [20]. 

In this present study, the analysis of TMEM176B protein expression in lung adenocarcinoma was examined with relevant clinical and pathological data. The findings demonstrated a notable increase in TMEM176B expression in tumour tissues compared to their corresponding adjacent normal tissues. Moreover, patients exhibiting higher TMEM176B expression demonstrated a comparatively reduced overall survival period in contrast to those with lower expression levels. Our study demonstrated the important role played by TMEM176B in lung adenocarcinoma cells. The overexpression of TMEM176B in both PC9 and A549 cell lines led to an increase in vitro cell proliferation, invasion, migration and adhesion. Recently, a study suggested that the AKT/mTOR signalling pathway is a major pathway in TMEM176B-dependent signalling in triple-negative breast cancer [18]. However, in our present study, the administration of AKT or PI3K inhibitors did not yield a significant impact on cell proliferation. Interestingly, the promotion of cell proliferation was diminished in both PC9 and A549 cell lines when treated with a JNK inhibitor. This trend was consistent across various cell functions, including invasion, migration and adhesion, upon treatment with the JNK inhibitor. These findings suggest that JNK serves as a crucial molecule in regulating cell functions in lung adenocarcinoma cells via the overexpression of TMEM176B.

According to the results of our co-immunoprecipitation experiment, an interaction between FGFR1 and TMEM176B was demonstrated. Cell function assays were conducted using the FGFR inhibitors Fexagratinib and Infigratinib. The outcomes indicated that both inhibitors were effective in attenuating the promoting trend, and some even reversed the trend. For instance, cell migration significantly decreased in both PC9 and A549 overexpression cells treated with Infigratinib compared to control cells. A study suggests that the FGFR1/JNK/MMP26 signalling cascade is a pivotal pathway in regulating cell invasion in NSCLC [27]. Another study demonstrated that high FGFR expression is associated with poor survival in uveal melanoma patients, and FGF9/FGFR/JNK or ERK signalling cascades are key pathways in regulating hepatic metastasis [28]. 

In our mass spectrometry experiment using immunoprecipitation samples, we identified 342 proteins that directly or indirectly interacted with TMEM176B. Among these proteins, Vimentin was notable, known for its significance in epithelial–mesenchymal transition (EMT). Studies have indicated that FGF9 activates FAK, AKT and ERK signalling through FGFR1, inducing EMT to stimulate tumourigenesis and hepatic metastasis in Lewis lung carcinoma cells [29]. Additionally, bee venom suppresses EGF-induced EMT in A549 cells by inhibiting the phosphorylation of ERK, FAK and mTOR and consequently reducing the expression of ZEB2 and Slug [30]. These findings suggest that ERK plays a crucial role in regulating EMT in non-small cell lung cancer (NSCLC). Furthermore, another study supported a mechanism wherein JNK plays a key role in regulating EMT in NSCLC; KLF4 inhibits metastasis and the migration of NSCLC via the TGFβ/JNK signalling pathway [31]. Our observations revealed that the overexpression of TMEM176B significantly upregulated JNK and phospho-JNK expression but not ERK. It also increased the expression of downstream EMT molecules, such as Vimentin and Snail. Importantly, these enhancements could be attenuated by the treatment of FGFR inhibitors.

Numerous targeted therapies, including those against VEGF, Kras, EGFR, ALK and immunotherapies such as PD1 and CTLA-4, have already gained approval for treating NSCLC (as reviewed in [32,33,34]). Owing to drug resistance and side effects, scientists continue to seek novel targets for NSCLC treatments. Identifying new targets could offer patients alternative therapies, minimise side effects and enhance their quality of life. Some emerging targets are currently undergoing clinical trials, such as ERK (NCT03520075), MET (NCT03539536, NCT04982224) and ACSS2 (NCT04990739). This underscores the necessity for developing new targets for NSCLC. Our study introduces a potential candidate, TMEM176B, as a new target. The research demonstrates its role in promoting lung adenocarcinoma development both in vitro and in vivo, suggesting that a TMEM176B inhibitor or neutralising antibody could be investigated for targeted therapy.

## 5. Conclusions

In summary, elevated TMEM176B expression was observed in lung adenocarcinoma, with higher levels associated with poorer prognosis and decreased overall survival. Moreover, the overexpression of TMEM176B led to increased cell proliferation, invasion, migration, and the adhesion of lung adenocarcinoma cells in vitro, and it also promoted tumour growth in vivo. The FGFR1/JNK/Vimentin/Snail signalling cascade emerges as a pivotal pathway governing EMT in lung adenocarcinoma through TMEM176B overexpression, and this suggests that TMEM176B is a potential therapeutic target for lung adenocarcinoma.

## Data Availability

All data generated and analysed during this study are available from the corresponding author upon reasonable request.

## Supplementary Materials

The following supporting information can be downloaded at https://www.mdpi.com/article/10.3390/cancers16132447/s1, Figure S1: There is no effect on ERK and Slug following overexpression of TMEM176B; Table S1: Patients’ detail of tissue array; Table S2: List of antibodies and Table S3: List of proteins interacting with TMEM176B.
