# Supplementary material for: TMEM176B Promotes EMT via FGFR/JNK Signalling in Development and Tumourigenesis of Lung Adenocarcinoma
Source: Cancers (Basel). 2024 Jul 3;16(13):2447. doi: 10.3390/cancers16132447 (PMC11240709; doi:10.3390/cancers16132447)
Supplement: Supplementary file 1 [file cancers-16-02447-s001.zip › Table S2 antiboby.pdf]

Table 1. Primary and secondary antibodies used in current study

Protein target	Cat. number	Supplier	Purpose
Rabbit anti-GAPDH	5174	CST	WB
Rabbit anti-LR8 (TMEM176B)	ab236860	Abcam	WB, IHC
Rabbit anti-TMEM176B	orb631961	Biorbyt	IP
Rabbit anti-JNK	9252	CST	WB
Rabbit anti-p-JNK	4668	CST	WB
Rabbit anti-ERK1/2	4695	CST	WB
Rabbit anti-p-ERK1/2	4370	CST	WB
Rabbit anti-FGFR1	9740	CST	WB
Rabbit anti-FGFR1	GB115541	Servicebio	IHC
Rabbit anti-VIM	5741	CST	WB
Rabbit anti-VIM	GB11192	Servicebio	IHC
Rabbit anti-Snail	3879	CST	WB
Rabbit anti-Slug	9585	CST	WB
Rabbit anti-E-cadherin	GB11082	Servicebio	IHC
Goat Anti-Rabbit IgG H&L (HRP)	ab6721	Abcam	WB, IHC
