# Supplementary figures and images for: TMEM176B Promotes EMT via FGFR/JNK Signalling in Development and Tumourigenesis of Lung Adenocarcinoma
Source: Cancers (Basel). 2024 Jul 3;16(13):2447. doi: 10.3390/cancers16132447 (PMC11240709; doi:10.3390/cancers16132447)

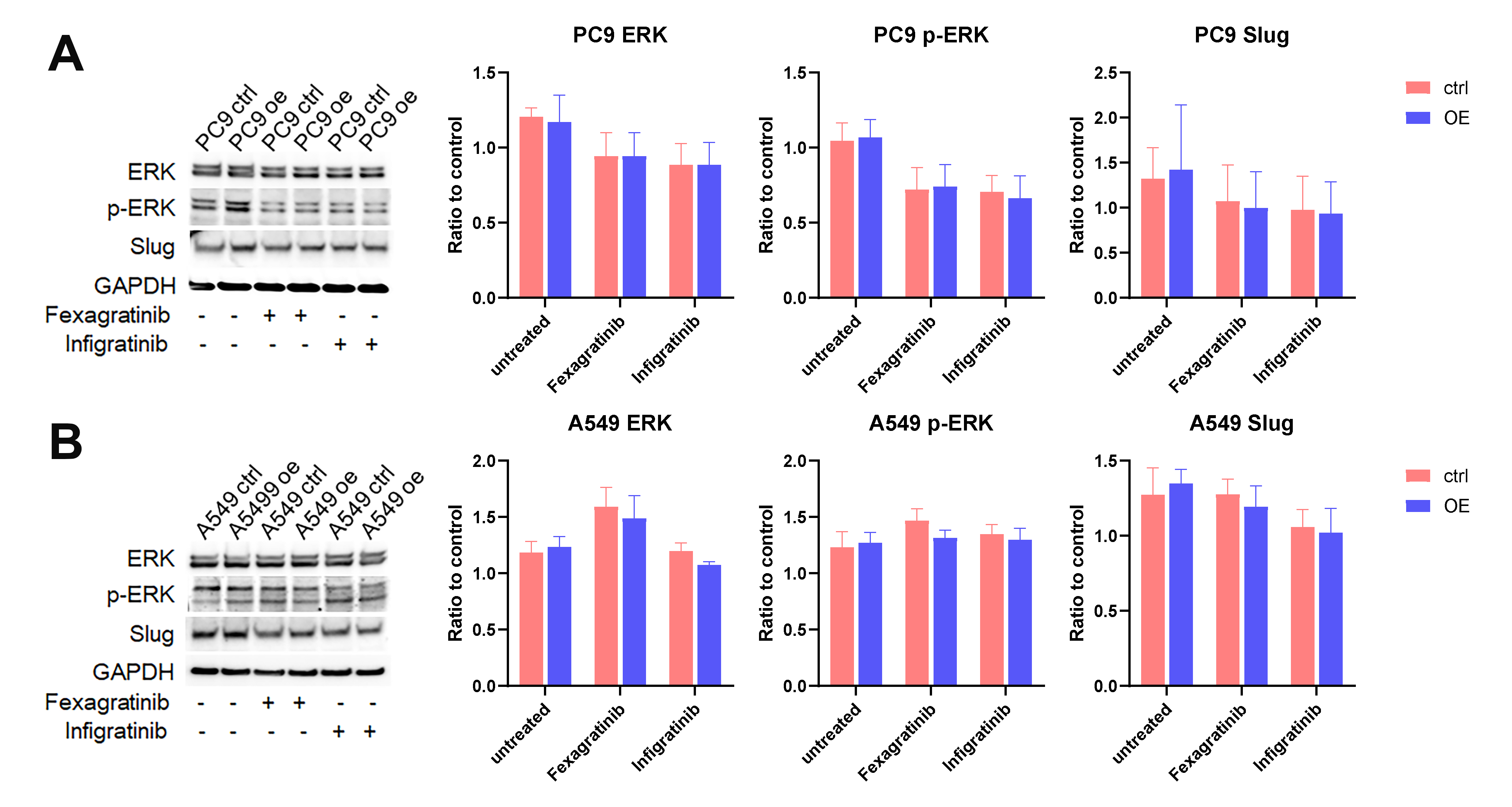

Supplement: Supplementary file 1 [file cancers-16-02447-s001.zip › Fig S1.tif]

Figure 2D

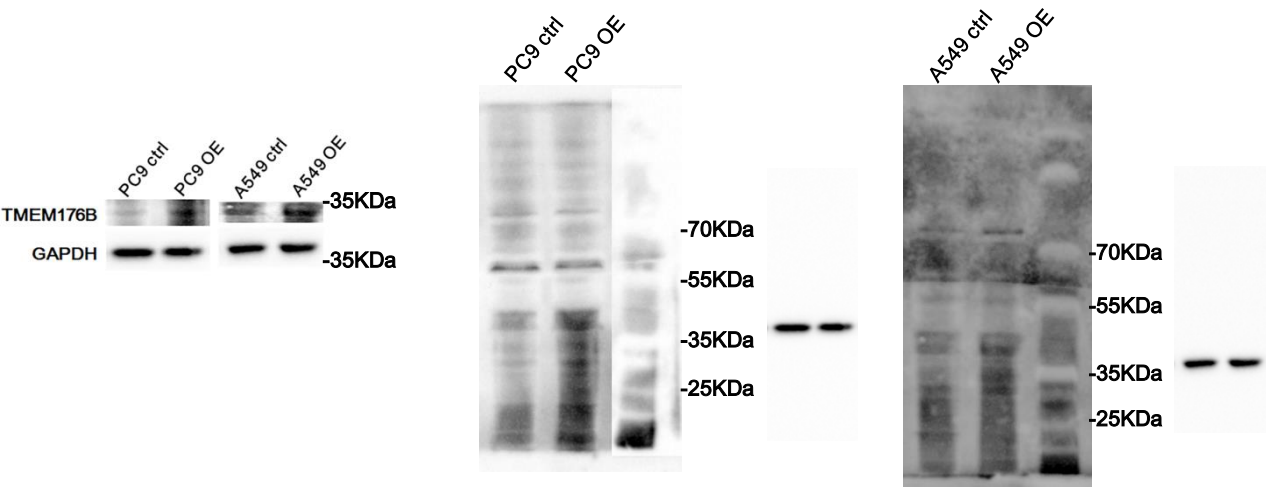

Figure 5A

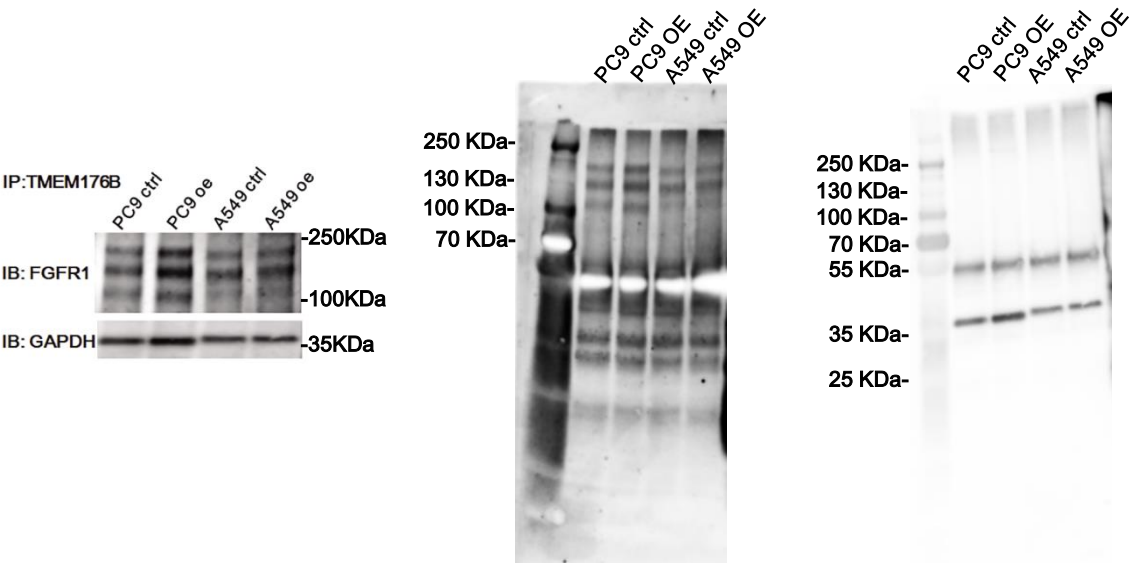

Figure 7A

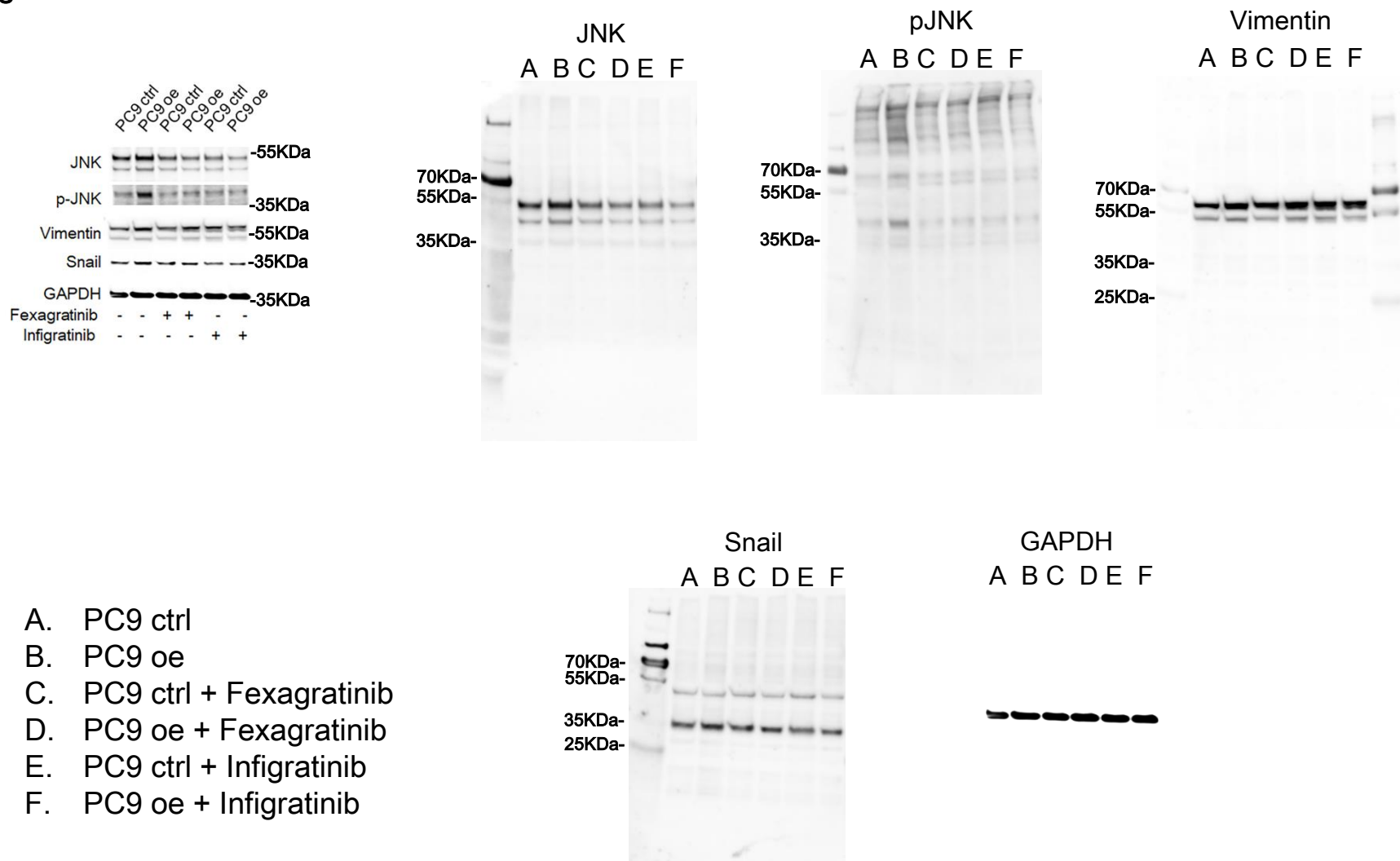

Figure 7B

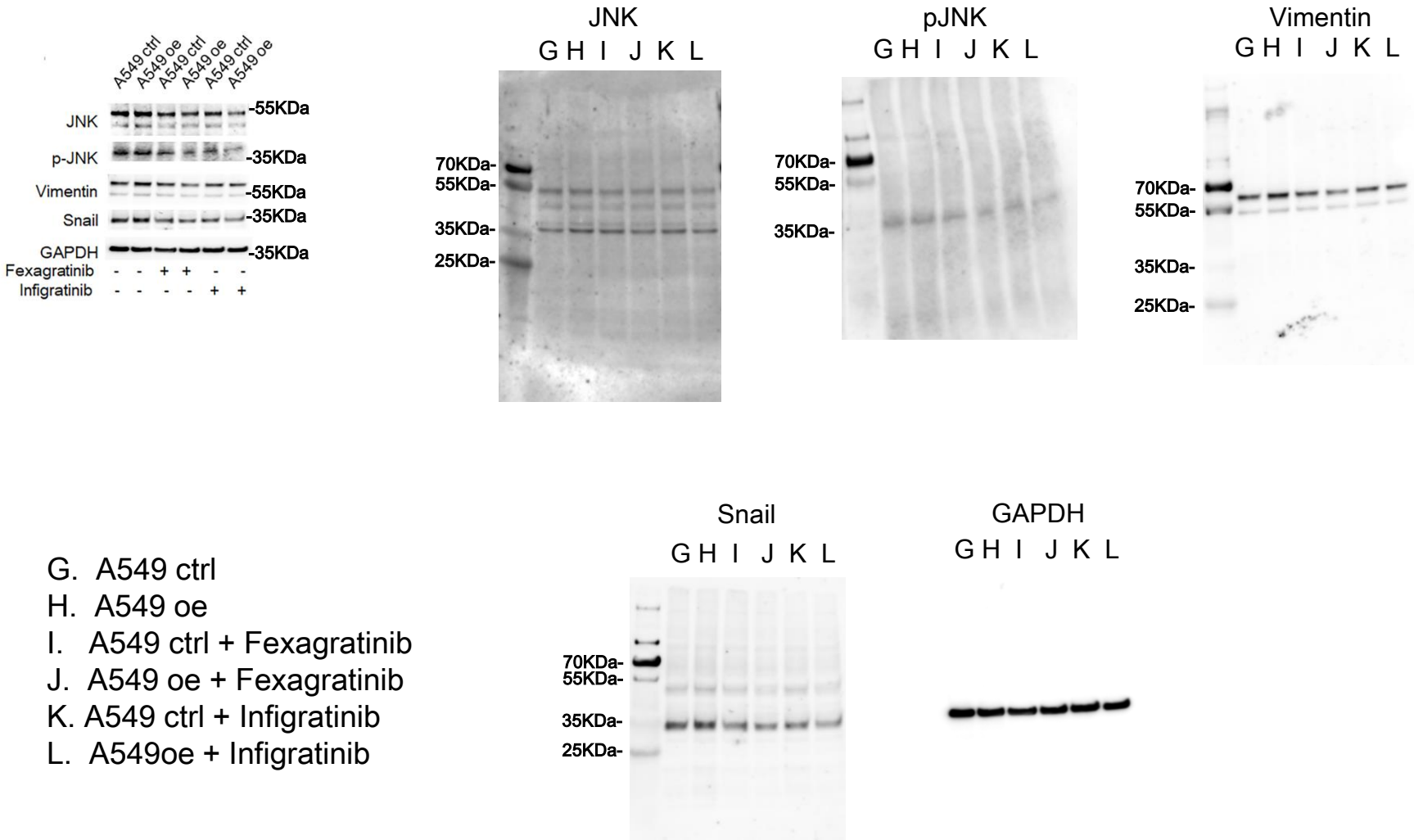

Figure S1A

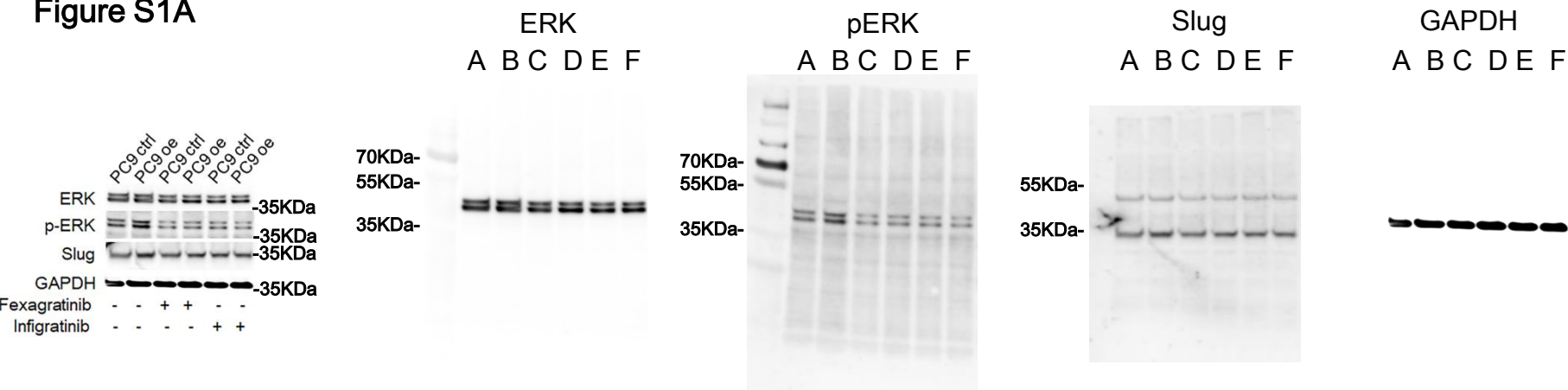

Figure S1B

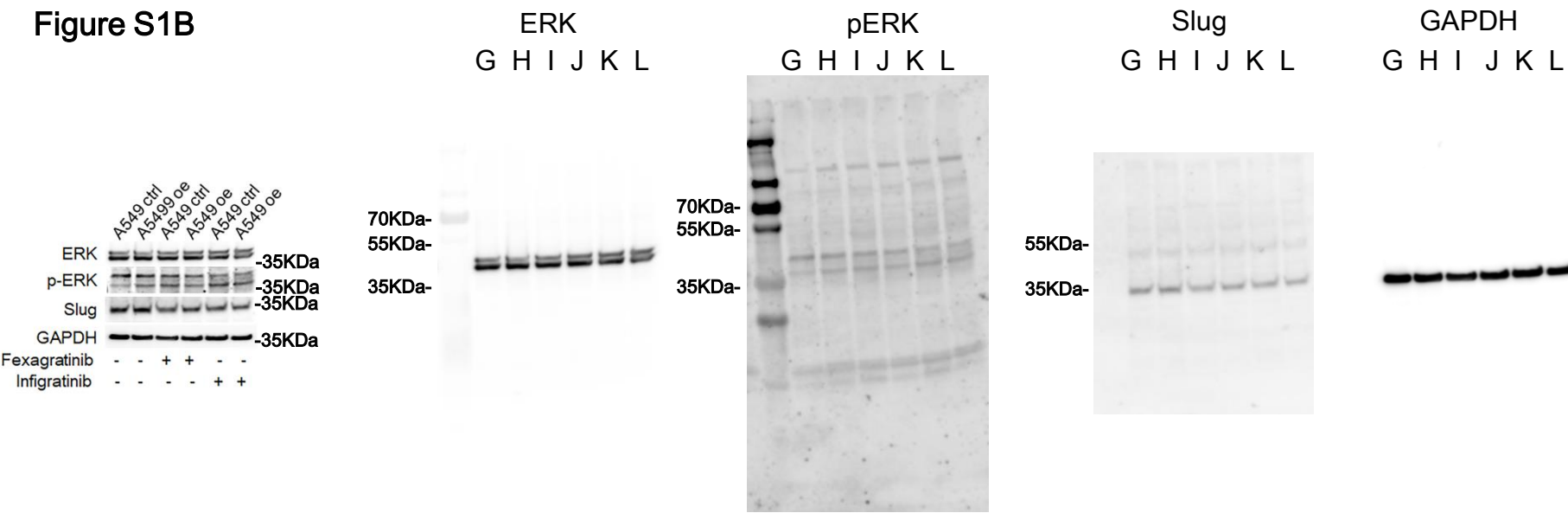

Supplement: Supplementary file 1 [file cancers-16-02447-s001.zip › WB.pdf]
